# The Differentially Regulated Cousins: Insights into the Differences in Transcriptional Regulatory Mechanisms Between HTLV-1 and HIV-1

**DOI:** 10.3390/v18010140

**Published:** 2026-01-22

**Authors:** Omnia Reda, Yorifumi Satou

**Affiliations:** 1 Division of Genomics and Transcriptomics, Joint Research Center for Human Retrovirus Infection, Kumamoto University, Kumamoto 860-0811, Japan; 2 Microbiology Department, High Institute of Public Health, Alexandria University, Alexandria 21615, Egypt

**Keywords:** *Retroviridae*, Retroviral transcriptional regulation, *Tax*, *Tax* bursts, *HBZ*, Tat, intragenic virus regulation, intragenic enhancer, bidirectional transcription, antisense transcription, HTLV-1 proviral silencing, HIV-1 latency

## Abstract

HTLV-1 and HIV-1 represent biologically significant, structurally close, and equally problematic yet divergent human retroviruses. Although both infect CD4+ T cells and share similar structural elements, they differ markedly in genomic stability, transmission dynamics, clinical progression, and, most importantly, their transcriptional regulatory mechanisms. HTLV-1, an ancient virus with a limited global burden, often remains asymptomatic for decades before potentially causing ATL or HAM/TSP. Conversely, HIV-1, a relatively recent zoonotic transmission, undergoes rapid replication, exhibits high genetic diversity, and causes progressive immunodeficiency unless controlled by antiretroviral therapy (ART). At the molecular level, HTLV-1 maintains proviral latency through a balanced bidirectional transcription of regulatory genes (e.g., *Tax* and *HBZ*) that manipulate host transcription and immune evasion pathways, facilitating persistence and oncogenesis. *HBZ* and *Tax* were shown to contribute to driving the progressive acquisition of Treg-like and HLA class II phenotype in chronically activated CD4+ T-cells, promoting tolerogenic antigen presentation and immune evasion in ATL cells. This well-controlled differential expression of HTLV-1 regulatory genes is attributed to multiple intragenic virus regulatory mechanisms, which will be discussed in this review. In contrast, HIV-1 transcription is driven by a tightly regulated 5′ LTR promoter involving host factors such as NF-κB, Sp1, AP-1, and NFAT, among others, with strong influence imposed by the landscape of the provirus integration site, playing a pivotal role in latency and reactivation. The distinct regulatory circuitry of each virus suggests a key difference in their essential regulation, with HTLV-1 primarily relying on intragenic mechanisms, while HIV-1 relies more heavily on interactions with the surrounding host environment to control its expression. This difference underscores unique therapeutic challenges in managing viral latency, persistence, and pathogenesis.

## 1. Introduction

The Retroviruses family (*Retroviridae* family), as the name implies, has a unique capability of reverse-transcribing its RNA and subsequently integrating the generated DNA into the host genome of the infected cell, taking great advantage of the host cell machinery to replicate. This family has eleven genera [1], three of which were classified to represent a *fossil* of historical human exogenous retroviruses, known as the Human Endogenous Retroviruses (HERVs) [2]. HERVs’ genetic elements occupy ~8% of the human genome. Over millions of years, this group of retroviruses has gradually diminished its ability to replicate; however, it has been associated with numerous pathological conditions both solely and due to activation/induction by exogenous retrovirus infection [3,4,5,6]. This effect is not surprising, given that ~320,000 HERV-related transcription factor binding sites (TFBSs) were identified [7], explaining the important role played by HERVs in the regulation of human genes.

The *Deltaretrovirus* and *Lentivirus* genera represent the exogenous retroviruses of the *Retroviridae* family, which includes human retroviruses, Human T-cell Leukemia Virus and Human Immunodeficiency Virus (HTLV) and (HIV), respectively. HTLV is a well-reported human pathogen and was the first retrovirus identified in humans in 1980 [8,9]. Several types have also been identified, including HTLV-2, 3, and 4, where 1 and 2 are the most studied types. HTLV-2 was isolated from a hairy-cell leukemia patient; however, there is no definite confirmation of its etiological relation to lymphoproliferative disorders [10,11]. On the other hand, HIV was first isolated in 1983 from a lymph node of a homosexual patient with multiple lymphadenopathies that was thought to develop acquired Immune Deficiency Syndrome (AIDS) [12]. HIV-1 has two main types, namely 1 and 2, with the latter having restricted geographical distribution, lower infectivity, and slower disease progression [13].

Despite sharing a familial affiliation within the *Retroviridae* family and a common structural foundation of two single-stranded RNA genomes, HTLV-1 and HIV-1 diverge in both clinical course and molecular regulation. They also demonstrate distinct transmission histories and disease outcomes. A comparative summary of major differences between HTLV-1 and HIV-1 is provided in Table 1. In the first part of this review, we will discuss the biological and clinical aspects of both viruses. The final section is devoted to a detailed comparison of the regulatory mechanisms of HTLV-1 and HIV-1.

## 2. Zoonotic Emergence and Global Burden

HTLV-1 was first to appear more than 27 thousand years ago following STLV-1 cross-species transmission [14]. However, its global burden remains significantly lower than HIV-1, with approximately 10 million people infected compared to HIV-1’s 40 million [15,16,17]. HIV-1 is believed to have crossed into the human population relatively recently, between 1920 and 1940, as confirmed by phylogenetic and epidemiological data [18]. Moreover, HIV-1 exhibits a disproportionate, yet global distribution, with eastern and southern Africa being highly impacted regions [17]. HTLV-1 shows a more restricted endemicity in regions such as Japan, South America, sub-Saharan Africa, and central Australia [15,16,19,20]. Notably, in HTLV-1 hotspot regions in Japan, the incidence of peripheral T-cell lymphoma is approximately 80 cases per 100,000 infected individuals [21].

## 3. Determinants of HTLV-1 and HIV-1 Pathogenesis (Natural Course, Molecular and Cellular Basis of HTLV-1 and HIV-1 Pathogenesis)

### 3.1. Transmission, Target Cells, and Clinical Course

HTLV-1’s main routes of infection include breastfeeding, sexual intercourse, and blood transfusion [22,23]. CD4+ T cells and, to a limited extent, CD8+ T cells, as well as dendritic cells (DCs), are the primary targets [24,25,26].

HTLV-1 transmission is almost exclusively reliant on cell-to-cell transmission, with a low replication level in vivo, and little viremia in peripheral blood. This HTLV-1 cell-to-cell transmission relies primarily on the formation of virological synapses [27]. However, Pais-Correia et al. [28] described that HTLV-1 can also form biofilm-like extracellular viral assemblies composed of budding virions embedded in adhesive extracellular structures. These assemblies adhere to other lymphocytes, facilitating virus transmission and the initiation of new infections. These transmission modes are not mutually exclusive and may act together to promote viral spread. Additionally, HTLV-1 can exploit cellular conduits, such as tunneling nanotubes, for transmission [29]. A comparative review of HTLV-1 and HIV-1 transmission modes and their impact on virus spread is provided in [30]. The observed low viremia in the peripheral blood suggests that the HTLV-1 primarily relies on the division of existing infected T-cell clones for survival (i.e., clonal proliferation) [27,31,32,33,34,35].

Clinically, after primary infection, the HTLV-1 virus spreads rapidly, with the proviral load reaching a set point soon after initial spread, reflecting the equilibrium between viral replication and the host immune response [33]. HTLV-1 infection can remain asymptomatic for decades, with 5–10% of cases progressing to Adult T-cell Leukemia/Lymphoma (ATL), HTLV-1-associated myelopathy/tropical spastic paraparesis (HAM/TSP) [15,22,36,37,38,39,40]. To a lesser extent, infection-associated uveitis or pulmonary inflammation may occur [41,42,43]. Moreover, HTLV-1c has been reported to be strongly associated with chronic fatal pulmonary diseases both in human and humanized mouse models [44,45].

ATL is classified into two major subtypes: aggressive and indolent-type ATL. The aggressive subtype, which includes acute ATL and lymphoma-type ATL, is characterized by rapidly proliferating leukemic cells. In contrast, the indolent subtype, comprising chronic ATL and smoldering ATL, progresses more slowly [46]. Overall, ATL is subdivided into four clinical stages: acute, chronic, smoldering ATL, and lymphoma [46]. The oncogenic functions of the key viral-encoded regulatory genes, *Tax* and *HBZ*, primarily drive the disease outcome (discussed in Section 4.1.1 of this review). A hallmark of HTLV-1 infection is that the virus preserves its copy number during chronic infection not through the generation of free viral particles, but rather through clonal expansion and long-term persistence of infected T-cell clones [22,40].

*Tax* and *HBZ* functions create a striking difference between HIV-1 and HTLV-1, which is the direct effect of the HTLV-1 provirus on host CD4+ T-cell differentiation, activation, proliferation, and apoptosis, which ultimately leads to malignant transformation of infected cells [47,48,49,50]. Several mechanisms underlying the oncogenic effect of HTLV-1, mainly orchestrated by *Tax* and *HBZ*, have been described and comprehensively reviewed in [32,51,52]. In a recent publication from our group, Tan et al. (2021) [53] used novel single-cell RNA-seq and TCR-seq modalities to identify molecular pathways controlling the in vivo malignant transformation of infected T cells. The analysis revealed that HTLV-1-infected cells exist in a persistently and highly activated state. Through an HBZ-mediated effect, these cells were driven into the progressive acquisition of a Treg-like phenotype. Upregulation of HLA class II was induced by a Tax-mediated effect. These changes, thereby, are thought to enable ATL cells to function as tolerogenic, costimulation-deficient antigen-presenting cells that induce T cell anergy and facilitate immune evasion. Moreover, the accumulation of replicative mutations along the HTLV-1 infection time course plays a substantial role in HTLV-1-induced oncogenesis [54,55,56]. A novel epigenetic-microRNA loop in developing ATL was reported in [57], where Polycomb repressive Complex 2 (PRC2)-mediated miR-31 repression was found to suppress NIK-mediated suppression of NF-κB, leading to cancer cell survival.

On the other hand, HIV-1 is mainly transmitted through vaginal or anal sex, and less frequently via blood products and/or sharing needles [58]. Mucosal tissues (rectal or vaginal) are the primary sites of HIV transmission, followed by initial replication taking place in intraepithelial and subepithelial DCs, CD4+ T cells, and macrophages [59]. HIV-1 then targets CD4+ T cells (mainly those expressing both CD4 and CCR5 receptors) through receptor-mediated entry, followed by a well-characterized acute phase marked by peak viral transmission with subsequent gradual establishment of a viral set point, caused by the inefficient HIV-specific immune response, along with the drastic depletion of the CD4+ target T cells [60]. In the absence of anti-retroviral therapy, HIV-1 progresses through well-defined clinical stages, including acute, then chronic infection, which eventually leads to an immunodeficiency status known as AIDS, well-characterized by depletion of CD4+ T cells. Virus replication is highly active during the acute phase, with plasma viremia detectable throughout the course of the disease [60].

Latently infected reservoirs are established as early as three days to four weeks post-infection [61,62] in peripheral blood, lymphoid nodes, and gut-associated lymphoid tissues. HIV-1 maintains its persistence through clonal expansion of latently infected cells, capable of evading immune clearance, along with minimal residual replication in certain anatomical sites [63,64,65,66]. HIV-1–infected cells persist through a dynamic balance between a clonal expansion increase and an immune-mediated clearance by cytotoxic T lymphocytes and natural killer cells. Of note, over half of the latent reservoir population is maintained through clonal expansion [63,64,67]. The decay of these reservoirs follows a biphasic model, with an initial half-life of 44 months and eventual stabilization, which is thought to occur through a doubling time of approximately 23 years [68]. Even with early antiretroviral therapy (ART) initiation, as seen in perinatal cases, viral rebound has been documented [69]. Of note, less than 1% of HIV-1-positive patients can spontaneously achieve prolonged viral remission in the absence of therapy [70,71]. Thus, viral reservoirs remain a formidable barrier to eradication. The advent of ART has transformed disease prognosis by suppressing viral load and preventing opportunistic infections. However, ART is not cytotoxic on virally infected cells and can’t block the provirus expression or clonal expansion of infected cells [65,72]. The dynamics of clonal expansion of the HIV-1 reservoir are well-reviewed in [65].

The defining feature of HIV-1 infection is the progressive loss/depletion of CD4+ T cells and the disturbance of their homeostasis. This leads to gradual immune deterioration and eventual death without therapy [73]. AIDS-related malignancies are often attributed to reasons other than direct CD4+ T cell transformation by the HIV-1 provirus. These reasons include infection-related immunodeficiency, immunosuppression, and chronic inflammatory conditions [74]. The malfunctioning immune system exacerbates co-infection with HHV-8 and HPV and subsequently leads to neoplastic transformation. In addition, Tat and Nef proteins have been reported to contribute to alterations in the cellular function, potentially influencing pathways that have been implicated in the neoplastic process [75,76,77,78,79,80].

Of note, in 2007, a case report described a rare HIV-1-associated B-cell lymphoma, likely attributed to the upregulation of the *STAT3* gene by HIV-1 5′ defective provirus integration with a compensating 3′LTR function [81]. HIV-1 provirus integration in cancer-related genes enhances infected cells’ proliferation and was reported for the possible development of T cell lymphoma in [82]. Most recently, Ya-Chi Ho and colleagues [83] showed that BACH2-driven programming transforms gut T cells from short-lived, interferon-driven effectors into long-lived tissue-resident memory cells, creating a stable HIV-1 reservoir while simultaneously promoting exhaustion of HIV-specific CD8+ T cells.

### 3.2. Genetic Sequence, Mutations, and Quasispecies

The HTLV-1 genetic sequence is categorized into seven main genetic subtypes (a–g) distinguished by differences in the nucleotide sequences of the long terminal repeat (LTR) region [84]. The most common of these, the cosmopolitan subtype (a), is further divided into five subgroups: Transcontinental, Japanese, West African, North African, and Peruvian Black [85]. While the Transcontinental subgroup is found globally, the other subgroups exhibit specific geographical distributions [86]. Others include five African subtypes (b, d–g) and an Australo/Melanesian subtype (c), which is found in Central Australia and Oceania [20,87,88,89,90].

Despite the reported genetic stability of HTLV-1 in comparison to HIV [91], ATL cells are known to often harbor defective 5′LTR of integrated proviruses and preserved pX region [92,93,94,95]. Of note, HTLV-1 defective proviruses have been reported to fall under either of 2 categories: type 1 and type 2, in which there is a missing sequence between 5′ and 3′ LTRs or when lacking 5′ LTR, respectively [96,97]. In a recent study conducted by our team, Katsyua et al. (2019) used HTLV-1 DNA-capture-seq to analyze the full proviral DNA, integration sites, and clonal expansion of infected cells in naturally HTLV-1-infected individuals from a Japanese cohort [98]. We detected the 5′ LTR-defective proviruses as the most common not only in ATL clones but across all the studied clinical groups, including asymptomatic carriers and HAM/TSP patients. These defective strains were associated with greater clonal expansion, likely due to immune evasion. In addition, this study detected a novel type 3 defective provirus lacking 3′ LTR, which reflects the complex impact of in vivo infection on the genetic stability of the HTLV-1 provirus [98].

HIV-1 includes groups M, N, O, and P. The most prevalent of all is group M, which leads most infections worldwide. It is further subdivided into nine subtypes (A–D, F–H, J, and K) and five sub-subtypes (A1–A3, F1, and F2). Within each subtype, a range of genetic diversity of at least 8% exists. Inter-subtype genetic diversity can reach up to 40% [99]. In contrast to HTLV-1, HIV-1 can undergo very frequent mutation and recombination during the reverse transcription process. So far, HIV-1 subtypes have produced 96 circulating recombinant forms (CRFs) and several unique recombinant forms (URFs) that are disseminated worldwide [100,101]. Virus recombination adds to the complexity of drug resistance and immune escape that already make HIV-1 infection challenging.

Clinically, defective HIV-1 proviruses predominate. Burner and colleagues [102] reported that within the initial weeks of infection, HIV-1 defective proviruses accumulate rapidly and come to represent more than 93% of the total proviral pool, independent of how early ART is started. Most of these defects were internal deletions, followed by deletions within the 3′ half and 5′ half of the genome, respectively [102,103,104]. Notably, the Siliciano group [105] reported that prior to ART start, intact provirus CD4+ T cells outnumbered those with defective proviruses. In all cases, the pool of infected cells with defective proviruses was reported to retain the capability of clonal expansion for more than a decade in vivo [106]. Moreover, it showed a mono-phasic slower rate of decay in comparison to the pool of infected cells harbouring intact proviruses [105,107].

### 3.3. Integration Preferences and Implications

HTLV-1 can infect a wide range of cells, including T- and B-lymphocytes, dendritic cells, and monocytes. However, HTLV-1 can transform T-cells [108,109,110,111]. HTLV-1 provirus integration lacks a strong predominant integration feature. HTLV-1 favors transcriptionally active regions of the human genome, including gene-dense regions, CpG islands, and transcription start sites (TSS) during the initial phase of cell-to-cell dissemination and clinically during the natural progression from carrier status to ATL onset [112,113,114]. Additionally, HTLV-1 was also reported to preferentially integrate into genomic regions near binding sites for specific host transcription factors, namely STAT1, p53, HDAC6, as well as chromatin remodeling factor, BRG-1. These host genomic factors were thought to affect the proviral expression and subsequently have a selective effect on the clonal expansion process [115,116].

On the contrary, HTLV-1 proviruses integrated into transcriptionally repressive DNA regions, particularly within the acrocentric chromosomes (13, 14, 15, 21, and 22), have been reported to more readily establish persistent infected T-cell clones in vivo, likely due to their ability to evade immune clearance [114,117]. Notably, HTLV-1 integration into the transcriptionally repressive nucleolar periphery was reported to confer a survival benefit by minimizing immune recognition, underscoring nucleolar organization as a critical determinant of long-term HTLV-1+ clone survival [118].

HIV-1 proviruses reside mainly in long-lived resting memory CD4+ T cells [119]. Macrophages, microglia, and dendritic cells can also contribute to specific anatomical sanctuaries [120,121]. Upon closer examination of the preferred integration sites, HIV-1 integration is primarily determined by the chromatin reader and transcriptional co-factor Lens epithelium-derived growth factor (LEDGF/p75), which can recognize nucleosomes associated with actively transcribed genes and subsequently direct integration into active chromatin regions [122,123]. This strong non-random bias for integration in introns of actively transcribing gene locations and in proximity to transcription-associated histone marks has been consistently observed and reported from both in vitro infection models and in the peripheral blood of people living with HIV (PLWH) [124,125,126,127,128,129], and comprehensively reviewed in [130,131,132]. Within this persistent tendency of integration, certain “hot spots” showed a strong, statistically proven preference for integration [133]. HIV-1 provirus can integrate across all chromosomes; however, it preferentially selects gene-dense chromosomes 19,17, and 16 in HIV- infected individuals [118,127]. On the contrary, studies using the ALV system to examine integration patterns revealed the same preference but also indicated that high transcriptional activity can impede integration [134].

The HIV integration landscape has a profound impact on the provirus’s fate, including its expression and persistence. In terms of persistence of HIV-1-infected cells/clonal expansion, in vivo studies have reported an enrichment of integration from clonally expanded cells in introns near the transcription start site of cancer-related genes, most frequently but not exclusively *STAT5B*, *BACH2*, *MKL2*, *MYB*, *POU2F1*, and *IL2RB*. That pattern is thought to confer a promotion of cell survival [135,136,137], which may be mediated through post-integration insertional mutations, aberrant splicing, and aberrant chimeric expression [138,139]. The other arm, which counteracts persistence and clonal expansion, is the immune surveillance by CTL responses.

Chromosomal profiling of individual proviruses from PLWH on ART reported that intact proviruses were enriched in non-genic locations and positioned relatively distant from transcriptional start sites after long suppressive antiretroviral therapy [140,141]. This observation has supported the notion of negative selection against transcriptionally active proviruses by immune clearance. Interestingly, biological sex was found to influence the host immune responses and affect the shaping of the latent reservoir [142]. In the distinct subgroup of people living with HIV, termed ‘elite controllers,’ who maintain undetectable levels of viral replication without treatment, intact proviruses favored integrations in non-genic or pseudogenic locations that consist of dense heterochromatin, suggestive of the deep silencing of these proviruses [143].

## 4. Regulation of Transcription

### 4.1. HTLV-1 Regulation of Transcription

#### 4.1.1. HTLV-1 Promoter and Regulatory Genes

**Figure 1:**
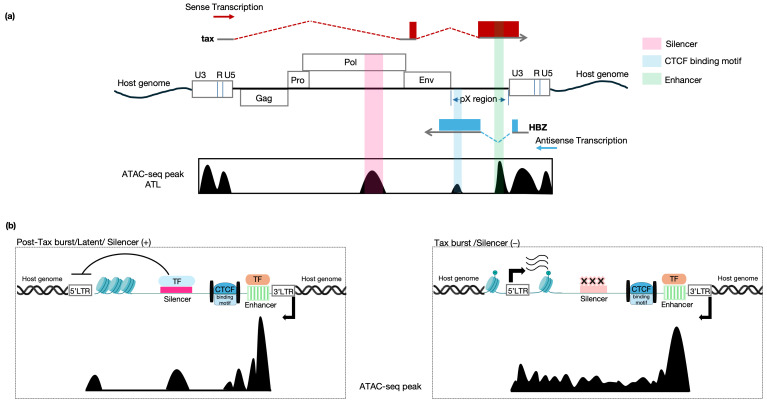
Major molecular mechanisms regulating HTLV-1 expression. (**a**) Schematic representation of the HTLV-1 provirus, with highlighted silencer region (pink), CTCF-binding site (blue), and enhancer region (green). Ssense and anti-sense transcription of regulatory proteins *Tax* and *HBZ* is shown, respectively. ATAC-seq peaksacrossn the provirus regiosn (5′ LTR, silencer, CTCF-binding site, enhancer, and 3′ LTR) from an ATL patient areindicatedn. (**b**) Schematic illustration of sense and anti-sense transcriptional regulation during the latent state (left panel) andduring *Tax* burst (right panel). At the latent steady state, silenced sensetranscriptionn is maintained through TFs binding to the silencer region. Uponnn extracellular stimuation s, the 5′LTR promoter dominates the silencer-mediatedrepression, induings a transientx transcriptional burs of Taxt. The anti-sense transcription persistsunder both conditions. Figure partially created in Biorender. Reda, O. (2026) https://BioRender.com/lo502hg.

The HTLV-1 viral genome consists of two copies of positive-sense RNA of approximately 9 kb in length. Upon cell entry, the retroviral ssRNA is reverse-transcribed into dsDNA and integrated into the host genome. The viral genome is flanked by long terminal repeats at the 5′ and the 3′ ends. The viral genome encodes several structural (*gag*, *pol*, *env*), regulatory (*tax* and *rex*), and accessory genes (*p12*, *p13*, *p30*, and *HTLV-1 bZIP factor* (*HBZ*)). Both LTRs carry key elements for transcription, polyadenylation, and integration [22]. Unlike HIV-1, HTLV-1 exhibits bidirectional transcription from both the 5′ and 3′ LTRs, both of which act as the promoters of the sense and antisense transcripts, respectively. Apart from *HBZ*, which is encoded from the anti-sense strand of the proviral genome, all other genes are encoded from the sense strand (Figure 1a).

*Tax* and *HBZ* are the key regulatory genes controlling the persistence and proliferation of HTLV-1-infected cells. *Tax* is thought to be expressed in intermittent bursts in vivo [144,145]. It strongly enhances sense-strand transcription through recruitment of CREB and coactivators CBP/p300 [146,147,148,149,150,151]. *Tax* functions are heavily dependent on *Tax*-mediated activation of NF-κB [152,153,154,155]. Functionally, *Tax* drives viral transcription, promotes cell cycle progression, T-cell immortalization, and anti-apoptotic signaling [145,156,157,158], yet paradoxically can also induce DNA damage, apoptosis, cell cycle arrest, and senescence owing to over-activation of NF-κB, ROS induction, or modulation of cyclin-dependent kinases [159,160,161]. Recent studies have revealed that HTLV-1 *Tax* expression in infected T cells occurs in intermittent bursts rather than being constitutively active. The Matsouka group [145] showed that these sporadic *Tax* on/off episodes occur in only a small fraction of cells at any given time, with individual bursts lasting approximately 19 h. Despite their brevity and low frequency, these transient bursts are sufficient to activate anti-apoptotic pathways, promoting the survival of the entire infected cell population. *Tax* expression can also be stress-induced and supports viral replication, highlighting a strategy for persistence and immune evasion. Most recently, the Bangham group [162] demonstrated that *Tax* bursts leave distinct imprints on the host cell: the immediate flare of *Tax* drives survival responses, whereas, as the burst subsides, a different transcriptional landscape emerges, including the rise in senescence markers and the persistence of pro-apoptotic signals. Together, these findings uncover a duality in *Tax*’s influence on cell survival and proliferation.

On the contrary, the in vivo constitutive transcription of *HBZ* [163] is critically driven by Sp1 and further augmented by JunD [164,165]. *HBZ* suppresses NF-κB activity and modulates host transcription factors such as CREB, c-JUN, JunD, JunB, and CBP/p300, thereby acting as a negative feedback regulator of *Tax*-driven transcription by binding the KIX domain of p300, which contributes to proviral latency [166,167], with small surges of *HBZ* expression appearing during late stages of *Tax* bursts [144]. As opposed to the highly immunogenic properties of the *Tax* protein, which is principally targeted by CTL responses [168,169,170,171,172], *HBZ* is low immunogenic and tolerated during immune surveillance [173,174].

HTLV-1, as a retrovirus, needs to keep a well-balanced equilibrium between the provirus latent state that can help infected cells to proliferate and escape the immune surveillance on one hand and the re-expression state that is mandatory for virus transmission to a new host cell. This notion can explain the difference and reciprocally regulated *Tax*/*HBZ* expression patterns. Current understanding reveals that the virus’s replicative genes, located on the plus-strand of the provirus, are activated in “sporadic, intense, self-limiting bursts” within individual cells [144,145]. In contrast, the minus strand of the integrated provirus, which encodes the *HBZ* gene, is stable [175].

#### 4.1.2. Intragenic Enhancer Region of HTLV-1

Recently, our group has reported a novel viral intragenic enhancer region within the HTLV-1 provirus genome [176] (Figure 1b). We identified the location of this previously uncharacterized region in the pX region near the 3′LTR promoter, particularly in the coding region of the *Tax* gene. The enhancer region significantly upregulated the anti-sense transcription by antagonizing the epigenetic silencing on the 3′LTR, producing the *HBZ* transcript. This activity is driven by the host transcription factors serum response factor (SRF) and Ets Like-1 protein (ELK-1), which bind to the nucleosome-free region (NFR), later identified as an intragenic enhancer. ChIP-seq signals corresponding to enhancer-associated histone modifications (H3K27Ac, H3K4me1, and H3K4me2) exhibited distinct peaks within 3 kilobases of the NFR, facilitating transcription factor binding and maintaining transcriptional activity. In addition, the enhancer activity induced aberrant transcription of host genes near the proviral integration site, potentially contributing to oncogenic transformation by dysregulating nearby cancer-related genes.

#### 4.1.3. Epigenetic Regulation of HTLV-1 Transcription

Epigenetic regulation also contributes to the dichotomy between sense and antisense transcription. The 5′ LTR is heavily methylated, silencing sense-strand transcription, while methylation does not extend across the entire provirus, leaving the 3′ LTR and *HBZ* gene unmethylated and transcriptionally active [177]. The first reported intragenic regulatory element of the HTLV-1 provirus was reported in [178] (Figure 1b). This study revealed that CTCF (an 11-zinc-finger protein highly conserved across species from flies to humans, and the most extensively studied insulator-binding protein in higher eukaryotes) binds to the HTLV-1 provirus, forming chromatin loops with the host genome that subsequently establishes an epigenetic boundary that prevents the spread of DNA methylation from the 5′ region toward the 3′ LTR. In an extended analysis from the same group [179], the HTLV-1 provirus was shown to form chromatin contacts in *cis* with the host genome as far as 1.4 Mb from the integration site, a good portion of which was dependent on CTCF binding. Given that HTLV-1 typically forms between 10^3^ and 10^5^ clones within an individual (each carrying the provirus integrated at a unique genomic site) [180], this study highlighted the clone-specific deregulation of host transcription resulting from abnormal chromatin interactions in *cis*. This effect is particularly significant considering the presence of tens of thousands of CTCF-binding sites across the human genome. In addition, these regulatory loops (chromatin insulators) were reported to disrupt host chromatin architecture, influencing both adjacent and distal gene expression relative to the provirus integration site.

Most recently, and adding to the previous discovery of an enhancer region within the HTLV-1 provirus, our team identified a novel silencer element within the provirus genome that regulates its latency by recruiting the RUNX1 transcriptional complex. The silencer region was identified as an open chromatin region (OCR) in the *pol* gene (polymerase coding region), which suppresses the 5′LTR promoter activity via RUNX-1 binding and subsequent recruitment of cofactors (CBFβ, HDAC3, Sin3A) to form a repressive complex. Of note, this silencer region was not found in HIV-1. Interestingly, single-cell multiome analysis in this study revealed that HTLV-1-infected cells oscillate between latent and burst states, with RUNX1 downregulation and ETS1 upregulation during bursts. These findings elucidated a key mechanism of HTLV-1 latency and answered the long-lasting question of the oscillating strand-selective transcription of HTLV-1 [181] (Figure 1b).

Together, these mechanisms (summarized and illustrated in Figure 1) explain the contrasting transcriptional kinetics of the two genes: *Tax* is expressed intermittently in rare, self-limiting bursts, whereas *HBZ* maintains continuous, low-level transcription. This reciprocal control allows HTLV-1 to balance replication with persistence; with transient *Tax* expression, driving viral propagation and cell proliferation, but limited by strong immune pressure, and with continuous *HBZ* expression supporting long-term survival and expansion of infected clones under immune surveillance.

#### 4.1.4. HIV-1 Regulation of Transcription (Figure 2)

**Figure 2:**
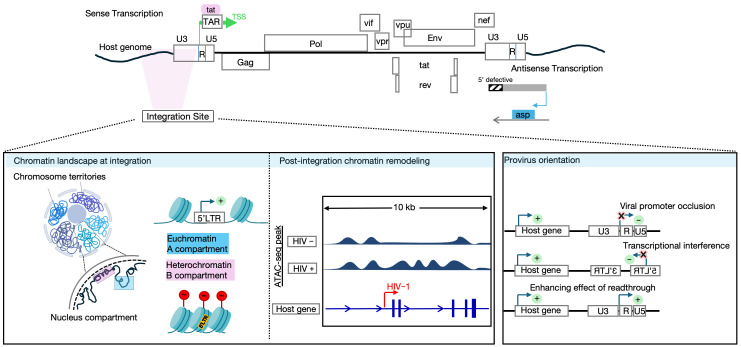
Regulation of HIV-1 expression. Upper panel: HIV-1 genome structure and transcription. HIV-1 sense transcription predominates over anti-sense transcription in all states, except for occasions of 5′ LTR deletions. *Cis*-regulatory elements at the 5′ LTR initiate transcription. Lower panel: Impact of the integration landscape on HIV-1 provirus transcriptional activity. Lower left panel: Effects of integration within distinct nuclear compartments. Integration into euchromatin region (Compartment A; closer to nuclear interior) is transcriptionally permissive, whereas integration into repressive chromatin regions (Compartment B; closer to the nuclear periphery) is associated with transcriptional repression. Lower middle panel: The HIV-1 promoter can override and remodel the chromatin state downstream of the integration site. Lower right panel: The orientation of proviral integration has a context-dependent effect on transcription. Integration in the same orientation as the host gene can result in viral promoter occlusion, whereas in other contexts it may promote transcriptional readthrough. Integration in the opposite orientation can cause transcriptional interference and repress proviral transcription or host-to-virus readthrough transcription. Figure partially created in Biorender. Reda, O. (2026) https://BioRender.com/repjb41.

Unlike HTLV-1, which relies on intragenic elements to control its expression, evade immune surveillance, and ensure its long-term survival, HIV-1 provirus expression is governed primarily by intertwined genetic and epigenetic features of the integration site, together with the state of the host cell and provirus intactness. These factors render provirus expression a stochastic phenomenon, often described as a “bet-hedging” strategy that helps the virus maintain its progeny [182,183,184]. This stochastic behavior was first evidenced by the observation that intact non-inducible proviruses do exist [103]. Secondly, this was supported by the sum of evidence that interrogating the latent cells with various latency-reversing agents or cell activators will only reverse latency in a fraction of cells at a single time point, regardless of the provirus inducibility [185,186], given the fact that HIV-1 gene expression is determined by the stochastic fluctuation of *Tat* expression [187,188] which has been additionally demonstrated to be sufficient to overcome cell-driven silencing of HIV transcription and the fundamental factor of viral autonomy regardless of the cell activation status [189]. Thirdly, chromatin environment at the integration site determines provirus transcription [190,191,192,193]. The Lichterfield group [141] profiled the epigenetic landscape of proviruses from PLWH on ART, and observed that the actively expressing proviruses are enriched in open chromatin regions, in comparison to transcriptionally silent proviruses that were found at chromatin repressive locations. In our team’s recent work on HIV-1, we engineered a dual-fluorescence reporter system [194] to better separate latent infected cells with a history of expression from those without a history of expression. We reported the integration preference of the infected cells with a history of expression near repressive histone marks, while those with no history of expression resembled the integration distribution of the readily expressing cells, both occurring near a permissive histone environment. The similarity and possible interchange between the two latter groups can be partially explained by these reported provirus–host chromatin interactions, but requires further analysis and additional proof. Lastly, HIV-1 proviral expression is often examined in relation to the transcriptional activity of the host gene in which it resides, and this influence appears to differ, though remains debated, depending on whether the provirus is integrated in the same or opposite orientation as the gene [195,196] (Figure 2, lower panel).

#### 4.1.5. HIV-1 Promoter, Enhancer Element, and Regulatory Genes

HIV-1 transcription is primarily unidirectional, originating from the 5′ LTR promoter. Its regulation involves nucleosome positioning (e.g., Nuc-0, Nuc-1), chromatin modifications, and interactions with host factors like LEDGF/p75 and CTCF [197,198].

The HIV-1 provirus coding regions are flanked by two identical LTRs located at the 5′ and the 3′ ends. In the case of HIV-1, the 5′LTR dominates and tightly controls the provirus transcription, functioning as the provirus promoter (Figure 2, upper panel). Functionally, it is subdivided into four regions: the modulatory, the enhancer, the core promoter, and the TAR region, demarcated by the TSS [199,200]. The former 3 regions comprise the U3 structural region, while the leader region constitutes the R and U5 structural end of the promoter. Interestingly, both the enhancer and the core promoter regions occupy a persistent nucleosome-free region expanding between 2 of the three nucleosomes well-deposited on the 5′LTR, namely Nuc-0 and Nuc-1 [201]. On the other hand, the 3′LTR controls the transcriptional termination and polyadenylation [202].

HIV-1 transcription proceeds through two main stages: The first is the initiation phase (Tat-independent stage), during which promoter activity is regulated by local chromatin environment (reviewed in [131,203,204]) and host cellular transcription factors. This is followed by a Tat-dependent stage, in which the viral Tat protein drives a robust transcriptional activation [124]. The initiation of transcription (in the absence of sufficient Tat or in a resting infected cell) is mainly dependent on the recruitment of cellular transcription factors to *cis*-acting elements within the promoter region. The key TFs that have been described in vivo to initiate HIV-transcription include NF-κB, NFAT, Sp1, and the TATA-box binding protein (TBP) [205,206,207,208], and reviewed in detail in [200,209,210,211,212,213]. In vitro studies have demonstrated a critical role of interferon regulatory factor-1 (IRF-1) as a co-regulator of early HIV-1 transcription by forming functional complexes with NF-κB (p50/p65) at κB binding sites within the 5′LTR enhancer (described below), thereby enhancing transcriptional output when Tat levels are limiting. This is accompanied by epigenetic modulations, as IRF-1 recruits histone acetyltransferases p300/CBP and P/CAF, while inhibition of histone deacetylases increases chromatin accessibility and LTR transcriptional competence [214,215,216].

If external stimuli are inducing provirus reactivation, cellular TFs aid a low-level resumption of transcription with subsequent Tat synthesis. With Tat accumulation, sufficient recruitment of cellular elongation machinery (the positive transcription elongation factor b (P-TEFb; CDK9-cyclin T1) to the nascent TAR element occurs [217], facilitating provirus transcription and elongation under what is known as the Tat positive feedback regulatory loop in which Tat acts as a multimodal transcriptional regulator operating through RNA-binding to TAR, host TF interaction, and the ability to override repressive chromatin environment through association with p300/CBP and P/CAF histone acetytransferases (HATs). Through these interactions, Tat enhances local histone acetylation and relieves chromatin-mediated repression at the HIV-1 long terminal repeat, facilitating sustained transcription from the integrated provirus [125,209,218,219,220,221,222,223,224,225,226].

Interestingly and relatively recently, Kuniholm et al. (2021) demonstrated that defective HIV-1 proviruses, which lack intact 5′ LTR promoters, can nevertheless remain transcriptionally active [227]. Using RT-ddPCR, 5′ RACE, reporter assays, and truncated proviral constructs, they showed that a substantial fraction of HIV-1 transcripts in resting CD4+ T cells, macrophages, and PBMCs from ART-treated individuals initiate within an intragenic region of the *env* gene (approximately nt 7760–8178, HXB2). These *env*-embedded *cis*-acting elements function as LTR-independent promoters, generating 5′ UTR-deficient transcripts [227].

#### 4.1.6. An Enhancer Function of HIV-1 Provirus

The HIV-promoter enhancer element spanning from −109 to −79 nucleotides with respect to the TSS is believed to act as a potent *cis*-regulatory element, given the presence of tandem repeats of NF-κB binding sites [216,228]. NF-κB, in turn, co-recruits additional cellular transcription factors to function effectively in activating provirus expression, most importantly Sp1 and AP-1, and critically IRF-1 during the Tat-independent phase of transcription [213,214,215,216].

A seminal study from Verdin and colleagues (1990) [229] identified a previously unrecognized *cis*-acting enhancer element within the coding region of the HIV-1 genome, distinct from the classical enhancer in the 5′ LTR. Using the HXB2 reference and reporter assays, they localized two discrete enhancer subdomains within the coding region of HIV-1: spanning nucleotides (nt) 4079–4342 and nt 4781–6026, encompassing the 3′ end of *pol*, the integrase coding region, and extending into *vif* and the first exon of *tat*. Both exhibited phorbol 12-myristate 13-acetate (PMA)-inducible enhancer activity on a heterologous herpes simplex virus thymidine kinase (HSV TK) promoter, independent of orientation or position, indicative of true enhancer function. This intragenic enhancer demonstrated cell-type specificity and was active in specific cell lines, suggesting distinct regulatory potential beyond the canonical LTR elements. Chromatin analyses of integrated proviruses revealed a cell-type-dependent, rather than universal, DNase I-hypersensitive site (HS7) (spanning nt 4534-4733, HXB2 isolate) within the potential intragenic enhancer element [230].

In a following study series from Verdin and Van Lint, they performed molecular characterization of this potential intragenic enhancer. They first demonstrated that the previously recognized intragenic regulatory element subdomain spanning from (nt 4079–4342, HXB2 isolate) contains three conserved AP-1 consensus motifs capable of sequence-specific binding by AP-1 proteins in a phorbol ester-inducible manner and that these motifs confer enhancer activity when positioned upstream of a heterologous promoter, establishing that internal proviral sequences can function as inducible regulatory elements [231]. They further identified the specific AP-1 family members c-Fos, JunB, and JunD as the primary proteins binding the three motifs. They additionally showed that these AP-1 sites are functionally required for optimal HIV-1 transcription and replication, as targeted mutation of one or more motifs diminishes enhancer activity, reduces viral gene expression, and impairs replication across multiple cell types, including T cells, monocytic cells, and primary macrophages [232,233].

Further high-resolution mapping of the initially identified intragenic enhancer region, they identified a nucleosome-free region centered around nt 4490 to 4766, with a ~500-bp nucleosome-free intragenic region in *pol* (nt 4481–4982). Within this module, four functional transcription factor binding sites were characterized: a GC-box bound by Sp1/Sp3, overlapping motifs recognized by ubiquitous and lymphoid nuclear proteins, and a PU.1/Spi-1 site mediating myeloid-specific regulation [234].

A very recent study from the Ho group [235] (Figure 2; lower panel)suggested that the HIV-1 provirus acts as an ectopic enhancer that increases local host chromatin accessibility, which in turn relies on local *cis*-regulatory elements (such as ZF, ETS, and RUNX family transcription factors) rather than global chromatin interactions within the infected cell. These findings build on earlier work showing that activation of HIV-1 transcription increases local chromatin accessibility downstream of the integration site, indicating that the integrated provirus can locally remodel host chromatin in *cis* [236]. Collora et al. further demonstrated the induction of chromatin looping between HIV-1 and host chromatin beyond the integration transcription unit and up to 100–300 kb away from the integration site [235]. Of note, in a preceding study [237], using a replication-competent in vivo infection model in a humanized mouse model, a KMT2A-mediated H3K4me3 modification was suggested to occur and positively impact efficient viral production. These observations come in line with and can partially explain the described HIV-1 provirus stochastic expression.

#### 4.1.7. Epigenetic Regulation of HIV-1 Transcription

Through looking deeply into the chromatin organization of the HIV-1 5′LTR. The core promoter region of HIV-1 is further subdivided into three regions, nuc-0, nuc-1, and nuc-2 [191,201]. They all play an important role in regulating the viral transcription. Nuc-0 is positioned upstream of the TSS, while Nuc-1 is located immediately downstream. These nucleosomes flank DNase I hypersensitive sites HSII and HSIII, which are regions of increased chromatin accessibility [230]. Under latent conditions, the positioning of Nuc-1, maintained by the BRG1- or HBRM-associated factor (BAF) complex, acts as a barrier to transcriptional elongation. Upon activation, chromatin remodeling leads to the repositioning or eviction of Nuc-1, facilitating transcriptional elongation and viral gene expression. This dynamic regulation of nucleosome positioning is crucial for the control of HIV-1 latency and reactivation [198,238].

In addition, a recent study observed that in the case of the unintegrated HIV-1 DNA, Nuc-0 and Nuc-2 are positioned slightly upstream compared to their locations in integrated proviral DNA. Upon integration into the host genome, these nucleosomes shift downstream to their canonical positions, a process referred to as nucleosome sliding. This repositioning is accompanied by the eviction of an additional nucleosome, NucDHS, which occupies a DNase hypersensitive site between Nuc-0 and Nuc-1 in unintegrated DNA. The sliding of Nuc-0 and Nuc-2, along with the removal of NucDHS, correlates with the activation of HIV-1 transcription, suggesting that these chromatin changes facilitate the transition from a repressive to an active transcriptional state. Notably, these nucleosome dynamics were observed in Jurkat T cells but not in primary CD4+ T cells, indicating cell-type-specific differences in chromatin remodeling during HIV-1 integration [238].

HIV-1 provirus sequence doesn’t have a CTCF binding site [178]; however, it was reported that CTCF binding sites are significantly enriched in regions of reduced chromatin accessibility in latently infected CD4+ T cells, indicating their role in maintaining HIV latency through chromatin insulation. Experimental knockdown or depletion of CTCF via shRNA or CRISPR-Cas9 led to increased HIV gene expression, directly linking CTCF to repression of viral transcription. Additionally, latency-reversing agents altered chromatin accessibility at CTCF-associated regions, suggesting that these sites are responsive to reactivation strategies. Overall, CTCF has been suggested as a key epigenetic regulator contributing to the maintenance of HIV latency [197].

Interestingly, transcription of the HIV-1 provirus was reported to induce the formation of a gene loop that brings the 5′ and 3′ LTRs into close physical proximity. The looping is dependent on active transcription and requires recognition of pre-mRNA processing signals, particularly the polyadenylation (poly(A)) signal and the 5′ splice donor site. Disruption of these RNA processing elements abolished loop formation, indicating that proper RNA processing is essential for maintaining this proviral architecture. The presence of the loop enhances transcriptional reinitiation, suggesting a functional role in sustaining high levels of HIV-1 gene expression. These findings reveal that LTR looping serves as a regulatory mechanism in HIV-1 transcription and may present a novel target for therapeutic intervention aimed at disrupting viral gene expression [239].

#### 4.1.8. The Role of Anti-Sense Transcription in HIV-1

The role of antisense transcription of the HIV-1 provirus, although initially underestimated, has emerged as a contributor to latency and viral persistence, particularly through the expression of antisense proteins like ASP and regulatory lncRNAs [213,240].

Klaver and Berkhout (1994) [241] explored the transcriptional activity of the HIV-1 3′ LTR, which functions as a promoter for antisense transcription. They demonstrated that the 3′LTR is capable of initiating transcription in the opposite direction relative to the viral sense genes. This antisense transcription is regulated by the same transcription factors that activate the sense 5′LTR, including Sp1 and NF-κB. Furthermore, the study revealed that the deletion of the 5′ LTR led to an upregulation of antisense transcription, suggesting a reciprocal regulatory relationship between the sense and antisense transcriptional processes. In addition, HIV-1 anti-sense transcript (AST) was reported to recruit the chromatin remodeling complex PRC2 to the 5′LTR and eventually leads to the establishment and maintenance of latency [242]. Most recently, in an overexpression experiment of anti-sense transcript in CD4+ T cells isolated from people living with HIV under suppressive anti-retroviral therapy, AST reduced the ability of provirus reactivation by latency reversing agents or T-cell stimulants [243]. The ratio of sense versus antisense transcriptional activity of HIV-1 promoters was assessed in in vitro studies and reported to be on average 100–2500 times less abundant than sense transcription [244,245]. The HIV-1 antisense transcript is expressed from a Tat-independent negative-sense promoter within the 3′LTR that potentially encodes a 189-amino acid protein, termed the antisense protein (ASP) [246,247,248] (Figure 2, upper panel).

Investigating the role of long noncoding RNA (asRNA) in regulating viral transcription, Saayman et al. (2014) [249] and Zapata et al. (2017) [242] reported its role in influencing the epigenetic landscape of the HIV-1 5′ promoter region, leading to the recruitment of repressive chromatin marks through association with components of the PRC2 complex, trimethylation of H3K27, Nuc-1 assembly, and subsequently suppressing viral gene expression.

## 5. Conclusions

HTLV-1 and HIV-1 illustrate how related human retroviruses can achieve persistence through fundamentally different regulatory strategies. The 5′ and 3′ LTR promoters are key regulators for HTLV-1 expression. However, HTLV-1 uniquely relies on intragenic regulatory elements, including enhancers, silencers, and CTCF-dependent chromatin boundaries, to control its bidirectional transcription and to orchestrate the reciprocal expression of *Tax* and *HBZ*. This finely tuned circuitry supports clonal proliferation, reversible latency, and oncogenic transformation while limiting immune recognition.

By contrast, HIV-1 transcription is largely governed by the 5′ LTR promoter and its chromatin environment, with Tat-dependent positive feedback, nucleosome positioning, and host transcription factors (such as NF-κB and Sp1), collectively shaping latency and reactivation. These distinct regulatory logics imply different therapeutic entry points: targeting intragenic control elements and *Tax*/*HBZ* balance in HTLV-1, versus modulating LTR chromatin, Tat-driven transcription, and antisense-mediated repression in HIV-1. Comparative dissection of these mechanisms may reveal shared principles of retroviral persistence and identify virus-specific vulnerabilities that can be exploited to improve strategies for HTLV-1-associated diseases and HIV-1 cure efforts.

## Figures and Tables

**Table 1:** Comparative overview of general and regulatory features of HTLV and HIV.

	Human T-Cell Leukemia Virus	Human Immunodeficiency Virus
**Epidemiological aspects**		
Emergence in humans	Appeared > 27,000 years ago from STLV-1 cross-species transmission	Emerged between 1920–1940 (cross-species origin confirmed by phylogenetic data)
First human reported case	1980	1983
Global Burden	~10 million	~40 million
Geographical Distribution	Restricted endemic regions (Japan, South America, sub-Saharan Africa, central Australia)	Global distribution (highest prevalence in Eastern and Southern Africa)
**Taxonomy, classification, and subtype diversity**		
Viral Genus	*Deltaretrovirus*	*Lentivirus*
Major types	HTLV-1, HTLV-2 (also HTLV-3, HTLV-4)	HIV-1, HIV-2
Genetic Subtypes	7 main subtypes (a–g)	4 major groups (M, N, O, P) M is the most prevalent with nine subtypes (A–D, F–H, and K)
Most Common Subtype/Group	Cosmopolitan subtype (a)	Group M (most global infections)
**Transmission, Tropism, and Pathogenesis**		
Routes of Transmission	Breastfeeding, sexual intercourse, and blood transfusion	Vaginal or anal sex, blood products, shared needles
Mode of Viral Spread	Cell-to-cell transmission (almost exclusively)	Cell-free and cell-to-cell transmission
Initial infection target	Unknown	Gastro-intestinal and vaginal mucosal cells (dendritic cells, M and epithelial cells)
Cellular Tropism/Target cells	T- and B-lymphocytes, dendritic cells, monocytes, fibroblasts	CD4+ T cells, also macrophages, microglia, and dendritic cells)
Clinical Course	Usually asymptomatic for decades; 5–10% develop ATL or HAM/TSP	Without ART: Proceed from Flu-like acute phase to clinical latency for decades until AIDs stage.
Associated morbidities	Adult T-cell leukemia/lymphoma (ATL), myelopathy (HAM/TSP), uveitis, pulmonary inflammation	Acquired Immunodeficiency Syndrome (AIDS) with opportunistic infections
Cell Transformation	Transforms only T-cells	No cellular transformation; causes immune cell depletion, and AIDs-related malignancies.
**Structure and key regulatory mechanisms**		
Provirus Genome	~9 kb double-stranded DNA	~9.7 kb double-stranded DNA
Key Regulatory Genes	*Tax* (activator) and *HBZ* (repressor/modulator)/both oncogenic	*Tat* (positive feedback transcription activator) and *Rev*, *Nef* (auxiliary regulators)
Transcription Directionality	Bidirectional	Unidirectional
Promoter Activity (LTRs)	Both LTRs act as promoters (sense & antisense)	5′ LTR/sense transcription mainly
Antisense Transcription	Constant antisense (*HBZ*) expression is critical for latency and immune evasion	Significantly less abundant antisense transcription encoding ASP (some role in chromatin remodeling)
Enhancer Regions	3′LTR and intragenic enhancer in pX region near 3′ LTR	5′ LTR enhancer (−109 to −79 bp) with tandem NF-κB sites.
Silencer Elements	Silencer region in *pol* gene	No dedicated viral silencer is described.
CTCF binding site	Intragenic single CTCF binding site	Provirus itself lacks a CTCF site, but CTCF enriched in the surrounding chromatin of latent cells
Reciprocal Regulation of transcription	*Tax* bursts ↔ *HBZ* steady expression = feedback loop balancing replication and latency	5′ LTR activity is potent with reported upregulation of 3′LTR transcription in case of deleted 5′LTR
Virus Transcription Pattern	“Sporadic, intense, self-limiting bursts” of sense transcription; stable antisense *HBZ*	Stochastic activation controlled by Tat positive feedback loop; latency driven by chromatin and nucleosome barriers

## Data Availability

No new data were created or analyzed in this study. Data sharing is not applicable to this article.

## Abbreviations

The following abbreviations are used in this manuscript:

HERVs	Human Endogenous Retroviruses
TFBSs	Transcription Factor Binding Sites
HTLV	Human T-cell Leukemia Virus
HIV	Human Immunodeficiency Virus
AIDS	Acquired Immune Deficiency Syndrome
DCs	Dendritic cells
ATL	Adult T-cell Leukemia/Lymphoma
HAM/TSP	HTLV-1-associated myelopathy/tropical spastic paraparesis
PRC2	Polycomb Repressive Complex 2
ART	Antiretroviral therapy
LTR	Long terminal repeat
CRFs	Circulating recombinant forms
URFs	Unique recombinant forms
TSS	Transcription start sites
LEDGF/p75	Chromatin reader and transcriptional co-factor Lens epithelium-derived growth factor
PLWH	People living with HIV
*HBZ*	*HTLV-1 bZIP factor*
SRF	Serum response factor
ELK-1	Ets Like-1 protein
NFR	Nucleosome-Free region
OCR	Open chromatin region
TBP	TATA-box binding protein
IRF-1	Interferon Regulatory Factor-1
P-TEFb	The positive transcription elongation factor b
HATs	Histone acetyltransferases
PMA	phorbol 12-myristate 13-acetate
HSV TK	heterologous herpes simplex virus thymidine kinase promoter
HS7	hypersensitive site 7
BAF	HBRM-associated factor
AST	Anti-sense transcript
ASP	Anti-sense protein
